# Poly-3-hydroxybutyrate production from acetate by recombinant *Pseudomonas stutzeri* with blocked L-leucine catabolism and enhanced growth in acetate

**DOI:** 10.3389/fbioe.2023.1297431

**Published:** 2023-11-08

**Authors:** Jieni Zhu, Wei Liu, Mengjiao Wang, Haiyan Di, Chuanjuan Lü, Ping Xu, Chao Gao, Cuiqing Ma

**Affiliations:** ^1^ State Key Laboratory of Microbial Technology, Shandong University, Qingdao, China; ^2^ State Key Laboratory of Microbial Metabolism, Shanghai Jiao Tong University, Shanghai, China

**Keywords:** acetate, *Pseudomonas stutzeri*, L-leucine catabolism, poly-3-hydroxybutyrate, CO_2_

## Abstract

Acetate is a low-cost feedstock for the production of different bio-chemicals. Electrochemical reduction of CO_2_ into acetate and subsequent acetate fermentation is a promising method for transforming CO_2_ into value-added chemicals. However, the significant inhibitory effect of acetate on microbial growth remains a barrier for acetate-based biorefinery. In this study, the deletion of genes involved in L-leucine degradation was found to be beneficial for the growth of *Pseudomonas stutzeri* A1501 in acetate. *P. stutzeri* (Δ*pst_3217*), in which the hydroxymethylglutaryl-CoA lyase catalyzing *β*-hydroxy-*β*-methylglutaryl-CoA into acetyl-CoA and acetoacetate was deleted, grew faster than other mutants and exhibited increased tolerance to acetate. Then, the genes *phbCAB* from *Ralstonia eutropha* H16 for poly-3-hydroxybutyrate (PHB) biosynthesis were overexpressed in *P. stutzeri* (∆*pst_3217*) and the recombinant strain *P. stutzeri* (∆*pst_3217*-*phbCAB*) can accumulate 0.11 g L^−1^ PHB from commercial acetate. Importantly, *P. stutzeri* (∆*pst_3217*-*phbCAB*) can also use CO_2_-derived acetate to produce PHB and the accumulated PHB accounted for 5.42% (w/w) of dried cell weight of *P. stutzeri* (∆*pst_3217*-*phbCAB*).

## Introduction

Manufacturing of chemicals from sustainable and low-cost feedstocks through bioprocesses is necessary with declining availability of fossil resources and increasing public environmental concerns. However, most bioprocesses use carbon sources generated from starch or other carbohydrate containing substances, resulting in competition between chemicals production and food industry. Acetate is emerging as an inexpensive non-food feedstock which can be easily produced through metal catalyzed carbonylation of methanol (Forster, 1979). Importantly, it can also be generated by depolymerization of lignocellulosic biomass (Gong et al., 2022), fixation of syngas via the acetogenic Wood-Ljundahl pathway (Straub et al., 2014) and upgrading CO_2_ by electrolysis (Zheng et al., 2022) (Supplementary Figure S1). For example, Zheng et al. (2022) designed an efficient two-step electrolysis process for acetate production from CO_2_. An aqueous acetate solution with a high purify of 97% can be obtained.

During the microbial acetate utilization, acetate is firstly converted to acetyl-CoA via acetate kinase-phosphotransacetylase pathway and acetyl-CoA synthetase (Kim et al., 2021). Then, acetyl-CoA will be assimilated through the glyoxylate cycle and TCA cycle or transformed into chemicals through various metabolic pathways. In recent years, there have been many researches on biotransformation of acetate into various valuable chemicals such as succinic acid (Narisetty et al., 2022), glycolic acid (Yu et al., 2020), itaconic acid (Merkel et al., 2022), (*R*)-3-hydroxybutyric acid (Fei et al., 2021), and glucose (Zheng et al., 2022) (Supplementary Figure S1). However, acetate at a low concentration can inhibit microbial growth and reduce metabolic efficiency (Lasko et al., 2000; De Mey et al., 2007), and microbial acetate conversion is still rather challenging.

Enhancing the capability of microorganisms to assimilate acetate is essential for improving feasibility of bioprocesses based on acetate. Since acetyl-CoA is the key intermediate during acetate utilization, acetate kinase, phosphotransacetylase and acetyl-CoA synthetase are often overexpressed to increase the assimilation of acetate and its activation to acetyl-CoA (Lin et al., 2006; Song et al., 2018; Narisetty et al., 2022). For example, Song et al. (2018) overexpressed acetyl-CoA synthetase in *Escherichia coli* to make the recombinant strain grow with acetate and accumulate isobutanol. The tolerance and acetate utilization can also be enhanced by some exogenous nutrients (Roe et al., 2002; Sandoval et al., 2011). Roe et al. (2002) found that methionine addition obviously restored the growth of *E. coli* under acetate pressure. However, exogenous nutrients addition may also increase the cost of the bioprocesses and may have a negative effect on the production of the desired products.

Besides activation of acetate, acetyl-CoA can also be endogenously generated through various microbial metabolic pathways like pyruvate dehydrogenation, fatty acid degradation, amino acids catabolism and ketone bodies decomposition (Supplementary Figure S2). These endogenous pathways may interfere with the acetyl-CoA production from exogenous acetate and decrease the efficiency of acetate utilization. Here, we constructed a series of mutants of *Pseudomonas stutzeri* A1501 with defection in acetyl-CoA production through L-leucine metabolism. Interesting, some mutants like *P. stutzeri* (∆*pst_3217*) exhibited increased tolerance and growth with acetate. Then, the poly-3-hydroxybutyrate (PHB) biosynthesis genes *phbCAB* from *Ralstonia eutropha* H16 were overexpressed in *P. stutzeri* (∆*pst_3217*) and the recombinant strain *P. stutzeri* (∆*pst_3217*-*phbCAB*) can produce PHB from commercial acetate. In addition, CO_2_-derived acetate produced through electrolysis was also utilized and the recombinant strain accumulated 5.42% PHB of dried cell weight. These results suggested that interception of endogenous acetyl-CoA generation might be a positive approach to increase the efficiency of acetate assimilation and acetate-based biorefinery.

## Materials and methods

### Reagents

Sodium acetate was purchased from Sinopharm Chemical Reagent Co., Shanghai, China. 3-Hydroxybutyrate methyl ester (3-HBME) was purchased from Sigma-Aldrich (United States). DNA oligonucleotides and DNA polymerase were purchased from Vazyme Biotechnology Co., Ltd., Nanjing, China. Restriction endonuclease enzymes were purchase from ThermoFisher (United States). T5 exonuclease was purchased from New England Biolab, Beijing. Aqueous acetate solution derived from CO_2_ through electrolysis was a kindly gift from Professor Zhigang Geng in Department of Chemical Physics, University of Science and Technology of China. All the other chemicals were of analytical pure grade and commercially available.

### Strains and culture conditions

The bacterial strains and plasmids used in this work are listed in Supplementary Table S1. Luria-Bertani (LB) medium containing yeast extract (5 g L^−1^), tryptone (10 g L^−1^), and sodium chloride (10 g L^−1^) and AB minimal medium (Baginsky and Rodwell, 1966) containing KH_2_PO_4_ (2.26 g L^−1^), K_2_HPO_4_ (4.1 g L^−1^), NaH_2_PO_4_ (2.53 g L^−1^), Na_2_HPO_4_ (3.34 g L^−1^), and trace metals (1%) were used for culture of *E. coli* and *P. stutzeri* in this work. The trace metal solution consisted of MgSO_4_·7H_2_O (14.8 g L^−1^), FeSO_4_·7H_2_O (0.55 g L^−1^), MnSO_4_∙4H_2_O (0.045 g L^−1^), and H_2_SO_4_ (0.14 g L^−1^). LB medium or AB minimal medium containing acetate as the sole carbon source (pH was adjusted to 7.0 after acetate addition) was used to cultivate *P. stutzeri* A1501 and its derivatives at 30°C and 200 rpm. *E. coli* strains DH5*α* and HB101 were cultivated in LB medium at 37°C and 180 rpm.

### Gene knockout and overexpression in *P. stutzeri* A1501

The primers used in this study are listed in Supplementary Table S2. Genes of *P. stutzeri* A1501 were deleted using the pK18*mobsacB* system as described previously (Xiao et al., 2021). The homologous arms upstream and downstream of the *pst_3217* gene were amplified using primers uf-*pst_3217*/ur-*pst_3217* and df-*pst_3217*/dr-*pst_3217* (Supplementary Table S2), respectively. The upstream and downstream fragments of *pst_3217* were fused together via recombinant PCR with primers uf*-pst_3217*/dr-*pst_3217*. The fusion fragments containing EcoRI and BamHI restriction sites were cloned into pK18*mobsacB* by T5 exonuclease DNA assembly (TEDA) method (Xia et al., 2019) to generate plasmid pK18*mobsacB*-*pst_3217′*. The pK18*mobsacB*-*pst_3217′* was transferred into *P. stutzeri* A1501 by the tri-parental mating with the help of *E. coli* HB101 carrying pRK2013 plasmid. The single-crossover mutants with the integration of the plasmid pK18*mobsacB*-*pst_3217′* into the chromosome were screened from M9 medium plates containing 20 g L^−1^ trisodium citrate as the sole carbon source with 40 μg mL^−1^ tetracycline. The double-crossover cells were selected on LB agar plates containing 20% (w/v) sucrose. Other mutants of *P. stutzeri* A1501 were constructed by using the same procedure.

For the expression of *phbCAB* in *P. stutzeri* (Δ*pst_3217*), the *phbCAB* gene cluster was amplified from *R. eutropha* H16 genome using primers *phbCAB*-f/r (Supplementary Table S2) and the PCR product was cloned into BamHI and SmaI restriction sites of pBBR1MCS-2 by TEDA method (Xia et al., 2019) to generate the expression plasmid pBBR1MCS-2-*phbCAB*. The plasmid pBBR1MCS-2-*phbCAB* was transferred into *P. stutzeri* (Δ*pst_3217*) by electroporation for PHB synthesis. The empty vector pBBR1MCS-2 was also transferred into *P. stutzeri* (Δ*pst_3217*) as the control.

### Measurement of dry cell weight

The dry cell weight was determined as previously described (Aoyagi et al., 1992). The bacterial cultures were collected and centrifuged at 6,980 × g for 10 min, and washed with distilled water. The pellets were frozen at −80°C overnight and the freeze-dried cells, and weighed. The dry cell weight was calculated by formulas:

$$\text{Dry cell weight }\left(g\;L^{-1}\right)=\frac{Weighed\;cell}{Collected\;volume}$$

### PHB extraction and analysis

PHB produced by the recombinant *P. stutzeri* (∆*pst_3217-phbCAB*) was quantified according to the previously described method with some modifications (Lo et al., 2009). After shake-flask growth, the bacterial cultures were collected and centrifuged at 6,980 × g for 10 min. The pellets were frozen at −80°C overnight and the freeze-dried cells (30–50 mg) were used to extract PHB by esterification as follows: suspension of freeze-dried cells by 850 µL methanol, 150 µL H_2_SO_4_, and 1 mL chloroform and the mixture was incubated at 100°C for 2 h. After cooling, 1 mL deionized water was added. After mixing by vortex and then static stratification, the bottom layer was filtered and injected into a gas chromatography (GC) (Shimadzu, GC 2014c) using a capillary GC column (AT. SE-54; inside diameter, 0.32 mm; length, 30 m). The GC analysis procedure was as follows: the temperatures of the injector and detector were maintained at 250°C and 290°C, respectively; the oven temperature was initially maintained at 80°C for 1 min and raised to 120°C with a rate of 10°C min^−1^, then continuously ramped up to 160°C at a rate of 45°C min^−1^ and maintained for 5 min.

### Analysis of acetate

The concentration of acetate was quantified by a high-performance liquid chromatography (HPLC) system (Shimadzu LC-20AD) equipped with an Aminex HPX-87H column (300 × 7.8 mm) and a refractive index detector (RID). The samples were boiled at 105°C for 10 min and then centrifuged at 13,523 × g for 15 min. The supernatants were filtered by a 0.22 µm filter for HPLC analysis. The column temperature was maintained at 55°C and 10 mM H_2_SO_4_ was used as the mobile phase with a flow rate of 0.4 mL min^−1^.

### Bacterial growth model

The growth of *P. stutzeri* A1501 and its derivatives in the AB minimal medium with different concentrations of acetate was assayed by Automated Microbiology Growth Analysis Systems (FP-1100-C, Bioscreen, Finland). The equation of Gompertz revised by Zwietering et al. (1990) was employed to calculate parameters such as lag time (*λ*) and exponential growth rate (*R*) through Graphpad 7.0 as described.

## Results

### Hydroxymethylglutaryl-CoA lyase deletion promoted growth of *P. stutzeri* A1501 with acetate

**FIGURE 1:**
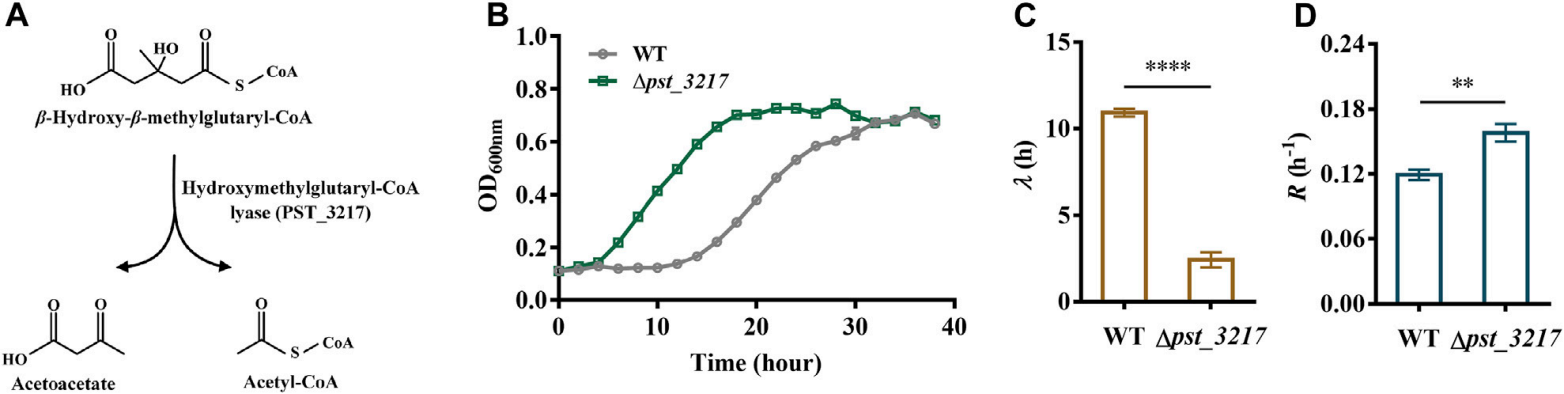
Deletion of *pst_3217* increased growth of *P. stutzeri* A1501 with acetate. **(A)** The reaction catalyzed by hydroxymethylglutaryl-CoA lyase (PST_3217). **(B)** Growth of *P. stutzeri* A1501 and *P. stutzeri* (∆*pst_3217*) in AB minimal medium with 40 mM acetate. **(C)** The lag time (*λ*) of *P. stutzeri* A1501 and *P. stutzeri* (∆*pst_3217*) in AB minimal medium with 40 mM acetate. **(D)** The exponential growth rate (*R*) of *P. stutzeri* A1501 and *P. stutzeri* (∆*pst_3217*) in AB minimal medium with 40 mM acetate. All data shown are the average values of three independent experiments.

Acetyl-CoA is an essential intermediate in microbial metabolism. It can be generated through acetate activation or different endogenous metabolic pathways (Supplementary Figure S2). Thus, blocking the endogenous acetyl-CoA generation might result in intracellular disbalance of acetyl-CoA and enhance the extracellular acetate utilization. The hydroxymethylglutaryl-CoA lyase in L-leucine catabolism pathway converts *β*-hydroxy-*β*-methylglutaryl-CoA to acetyl-CoA and acetoacetate (Figure 1A). Since directly deletion of the major acetyl-CoA generation pathway like pyruvate dehydrogenase *aceEF* may also induce severe growth defects (Da et al., 2021), the hydroxymethylglutaryl-CoA lyase encoding gene *pst_3217* was deleted in *P. stutzeri* A1501 to identify the feasibility of increasing acetate assimilation through interception of endogenous acetyl-CoA generation.

As shown in Figures 1B, C, *P. stutzeri* A1501 can grow using acetate as the sole carbon source with a long lag phase of 10.92 h. After *pst_3217* deletion, the lag time of *P. stutzeri* (∆*pst_3217*) decreased to 2.43 h. Importantly, deletion of *pst_3217* also increased the growth rate of *P. stutzeri* (∆*pst_3217*) with acetate. The exponential growth rate of *P. stutzeri* A1501 was 0.12 h^−1^ while the value of *P. stutzeri* (∆*pst_3217*) was 0.16 h^−1^ (Figure 1D). The growths of *P. stutzeri* A1501 and *P. stutzeri* (∆*pst_3217*) in AB minimal medium containing glucose and succinate were almost identical (Supplementary Figure S3). These results indicated that *pst_3217* deletion can specifically increase the growth of *P. stutzeri* A1501 with acetate.

### Mutation of other genes involved in L-leucine catabolism was beneficial for growth with acetate

**FIGURE 2:**
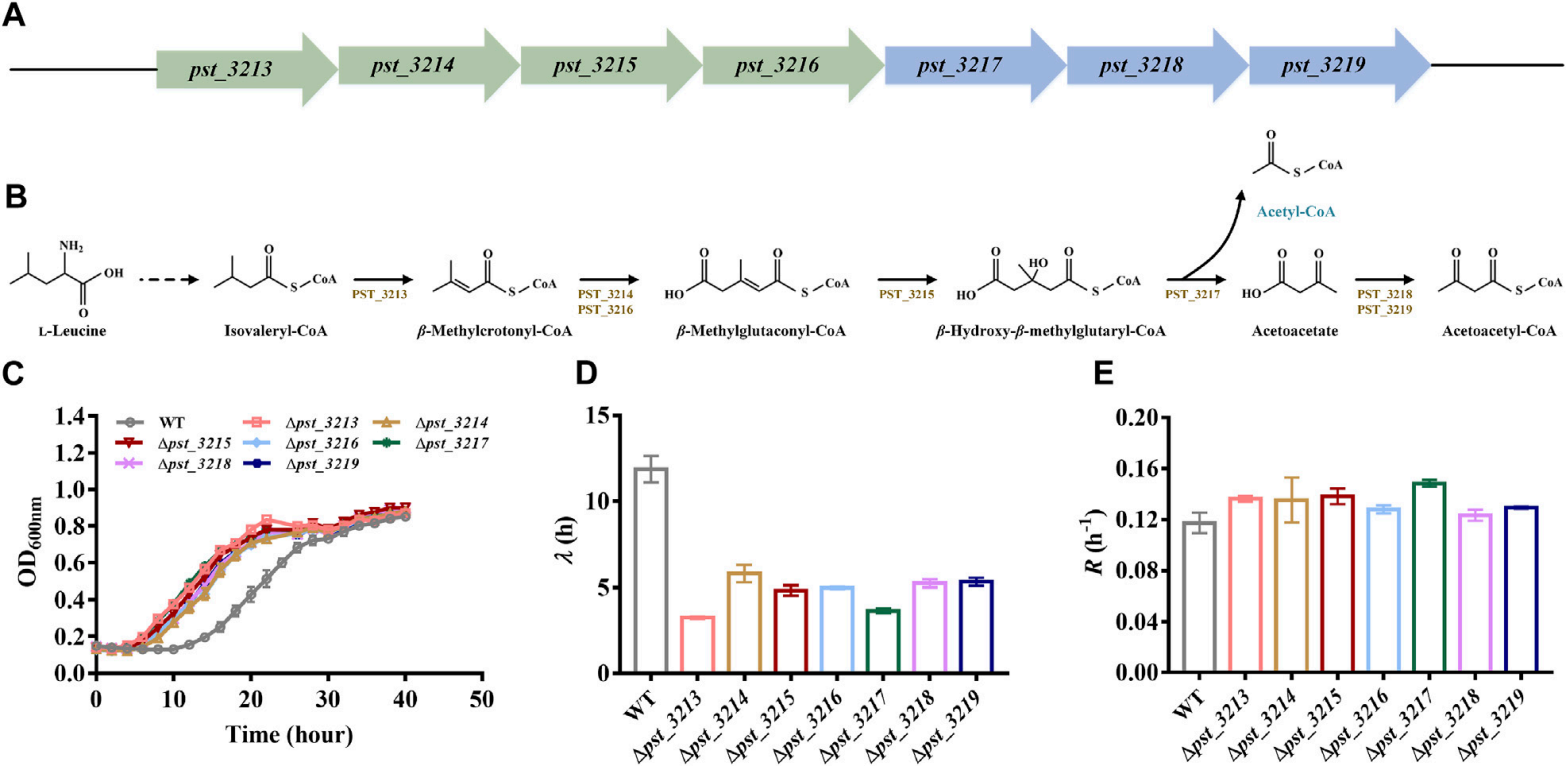
Deletion of genes involved in l-leucine catabolism was beneficial for growth of *P. stutzeri* A1501 with acetate. **(A)** The genes clusters involved in isovaleryl-CoA degradation of *P. stutzeri* A1501. **(B)** The catabolism pathway of l-leucine in *P. stutzeri* A1501. PST_3213, isovaleryl-CoA dehydrogenase; PST_3214, methylcrotonyl-CoA carboxylase, *α* subunit; PST_3215, *β*-methylglutaconyl-CoA hydratase; PST_3216, methylcrotonyl-CoA carboxylase, *β* subunit; PST_3217, hydroxymethylglutaryl-CoA lyase; PST_3218, CoA transferase, subunit A; PST_3219, CoA transferase, subunit B. **(C)** The growth of different strains in AB minimal medium with 40 mM acetate. **(D)** The lag time (*λ*) of different strains in AB minimal medium with 40 mM acetate. **(E)** The exponential growth rate (*R*) of different strains in AB minimal medium with 40 mM acetate.

The catabolism mechanism of L-leucine in *Pseudomonas* has been intensively studied. Branched-chain amino acid transaminases and branched-chain 2-keto acid dehydrogenase transform L-leucine into isovaleryl-CoA. Seven genes associated with isovaleryl-CoA degradation were annotated in genome of *P. stutzeri* A1501 (Figures 2A, B). Isovaleryl-CoA is eventually transformed into acetyl-CoA and acetoacetate via *pst_3213*, *pst_3214*, *pst_3215*, *pst_3216*, and *pst_3217*, while acetoacetate is catabolized into acetoacetyl-CoA by *pst_3218* and *pst_3219* and finally degraded to acetyl-CoA. Besides *pst_3217*, other genes involved in isovaleryl-CoA catabolism including *pst_3213, pst_3214*, *pst_3215*, *pst_3216*, *pst_3218*, and *pst_3219* were also deleted in *P. stutzeri* A1501.

Compared with *P. stutzeri* A1501, all of the mutants exhibited improved growth with shorter lag phases and higher growth rates in acetate (Figures 2C–E). Since *P. stutzeri* (∆*pst_3217*) grew faster than other obtained mutants, this strain was intensively studied in subsequent experiments.

### Deletion of hydroxymethylglutaryl-CoA lyase also improved the tolerance of *P. stutzeri* A1501 to acetate

**FIGURE 3:**
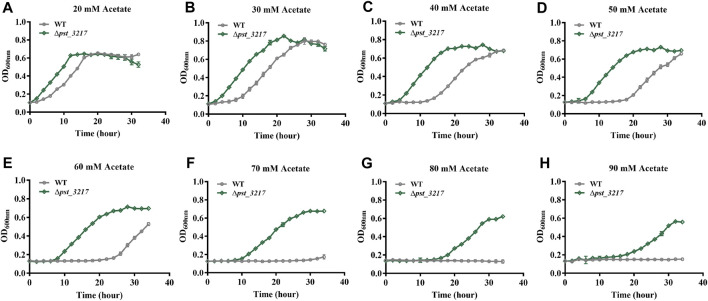
The growth of *P. stutzeri* A1501 and *P. stutzeri* (∆*pst_3217*) in AB minimal medium with different concentrations of acetate. **(A)** 20 mM acetate; **(B)** 30 mM acetate; **(C)** 40 mM acetate; **(D)** 50 mM acetate; **(E)** 60 mM acetate; **(F)** 70 mM acetate; **(G)** 80 mM acetate; **(H)** 90 mM acetate.

Acetate restrains microbial growth in a concentration-dependent manner. *P. stutzeri* (Δ*pst_3217*) exhibited increased growth in AB minimal medium with 40 mM acetate. The growth of the strain in AB minimal medium with different concentrations of acetate was also assayed by Automated Microbiology Growth Analysis Systems (FP-1100-C, Bioscreen, Finland). As shown in Figure 3, *P. stutzeri* A1501 was unable to grow in AB medium with acetate concentrations higher than 70 mM while obvious growth of *P. stutzeri* (∆*pst_3217*) can be observed in AB medium with 90 mM acetate. Compared with *P. stutzeri* A1501, *P. stutzeri* (∆*pst_3217*) exhibited faster growth rates and shorter lag times than those of *P. stutzeri* A1501 in the AB minimal medium with different concentrations of acetate. Thus, *P. stutzeri* (∆*pst_3217*) possessed tolerance to high concentrations of acetate.

### PHB production of *P. stutzeri* (∆*pst_3217-phbCAB*) from commercial acetate

**FIGURE 4:**
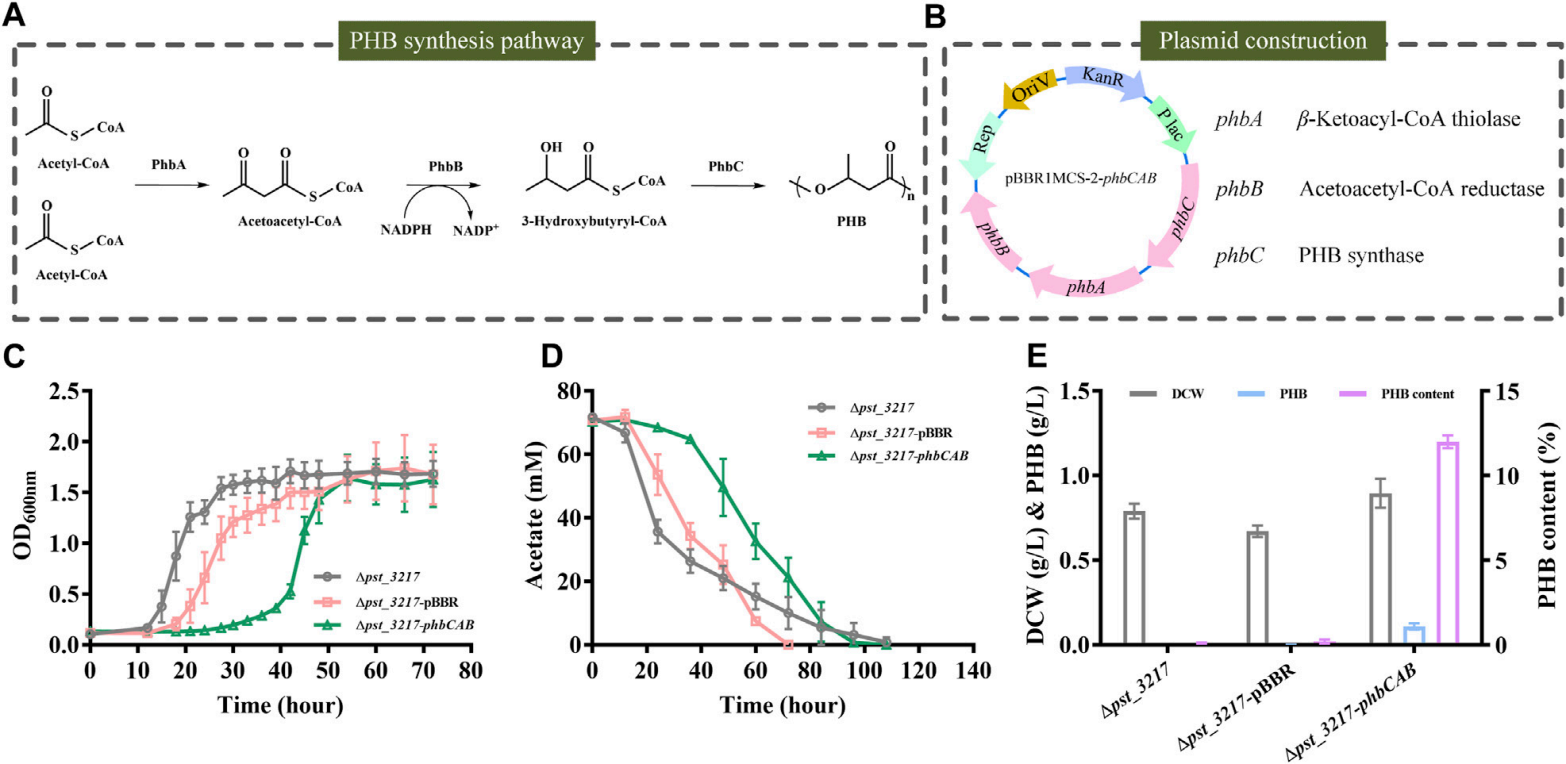
PHB production by *P. stutzeri* (∆*pst_3217*-*phbCAB*) from commercial acetate. **(A)** Schematic diagram of PHB biosynthesis pathway in *R. eutropha* H16. **(B)** The structure of plasmid pBBR1MCS-2-*phbCAB*. **(C)** Growth of *P. stutzeri* (∆*pst_3217*-*phbCAB*), *P. stutzeri* (∆*pst_3217-*pBBR) and *P. stutzeri* (∆*pst_3217*) in AB minimal medium with 70 mM commercial acetate. **(D)** Acetate consumption of *P. stutzeri* (∆*pst_3217*-*phbCAB*), *P. stutzeri* (∆*pst_3217-*pBBR) and *P. stutzeri* (∆*pst_3217*) in AB minimal medium with 70 mM commercial acetate. **(E)** Cell dry weight (DCW), PHB and PHB content of *P. stutzeri* (∆*pst_3217*-*phbCAB*), *P. stutzeri* (∆*pst_3217-*pBBR) and *P. stutzeri* (∆*pst_3217*) after cultivation in AB minimal medium with 70 mM commercial acetate.

Polyhydroxyalkanoates are microbiologically produced biodegradable plastics with flexible mechanical properties. PHB was the most extensively studied polyhydroxyalkanoates in recent years. The biosynthesis of PHB from acetyl-CoA comprises three enzymatic reactions catalyzed by *β*-ketoacyl-CoA thiolase (PhbA), acetoacetyl-CoA reductase (PhbB), and PHB synthase (PhbC) (Figure 4A).

The *phbCAB* genes were amplified from genome of *R. eutropha* H16, cloned into pBBR1MCS-2 and transferred into *P. stutzeri* (Δ*pst_3217*) (Figure 4B). Then, *P. stutzeri* (∆*pst_3217*-*phbCAB*), *P. stutzeri* (∆*pst_3217-*pBBR) and *P. stutzeri* (∆*pst_3217*) were cultivated in AB minimal medium with 70 mM commercial acetate. Gas chromatography analysis confirmed that *P. stutzeri* (∆*pst_3217*-*phbCAB*) can synthesize PHB from acetate (Supplementary Figure S4). As shown in Figures 4C, D, *P. stutzeri* (∆*pst_3217*-*phbCAB*) can grow in medium with 70 mM commercial acetate and accumulated 0.11 g L^−1^ PHB with a yield of 0.026 g g^−1^ acetate. The amount of PHB accounted for 12% (w/w) of dried cell weight of *P. stutzeri* (∆*pst_3217*-*phbCAB*) (Figure 4E). No accumulation of PHB was observed during the growth of *P. stutzeri* (∆*pst_3217-*pBBR) and *P. stutzeri* (∆*pst_3217*) (Figure 4E).

### PHB production from CO_2_-derived acetate

**FIGURE 5:**
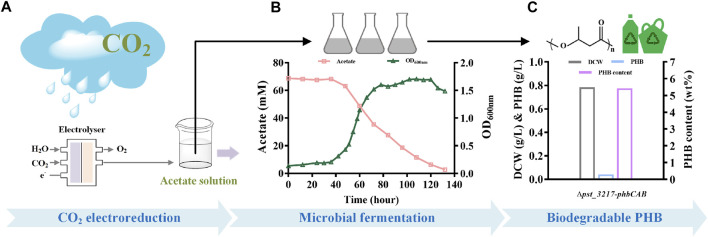
PHB production from CO_2_-derived acetate by *P. stutzeri* (∆*pst_3217*-*phbCAB*). **(A)** Scheme of acetate production through CO_2_ electroreduction. **(B)** Growth and acetate consumption of *P. stutzeri* (∆*pst_3217*-*phbCAB*) in medium with CO_2_-derived acetate. **(C)** Dry cell weight (DCW), PHB and PHB content of *P. stutzeri* (∆*pst_3217*-*phbCAB*) in medium with CO_2_-derived acetate.

To demonstrate the feasibility of upcycling CO_2_ into bioplastic, production of PHB from aqueous acetate solution derived from CO_2_ through electrolysis with *P. stutzeri* (∆*pst_3217*-*phbCAB*) was conducted (Figure 5). As shown in Figure 5B, *P. stutzeri* (∆*pst_3217*-*phbCAB*) can also grow with CO_2_-derived acetate as the carbon source. After culture at 30°C and 200 rpm for 132 h, the biomass of *P. stutzeri* (∆*pst_3217*-*phbCAB*) was close to that of in commercial acetate. PHB at a concentration of 42.45 mg L^−1^ was produced with a yield of 0.010 g g^−1^ acetate and the amount of PHB accounted for 5.42% (w/w) of dried cell weight of *P. stutzeri* (∆*pst_3217*-*phbCAB*) (Figure 5C).

## Discussion

Nowadays, there are 37 billion tons CO_2_ emitted into atmosphere every year (Zhang et al., 2023). Besides a key promotor of climate change, CO_2_ is also considered as a promising raw material in chemical industry. CO_2_ can be electro-reduced into methanol (Kattel et al., 2017; Zhang et al., 2023), formate (Stöckl et al., 2020; Xie et al., 2023) or acetate (Qiu et al., 2022; Zheng et al., 2022), and then transformed into PHB by different biotechnological routes. Herein, we discovered that blocking endogenous L-leucine degradation can increase the growth rate and tolerance of *P. stutzeri* A1501 in acetate. Then, PHB production from CO_2_-derived acetate by *P. stutzeri* (∆*pst_3217*-*phbCAB*) was conducted. PHB was finally accumulated at a concentration of 42.45 mg L^−1^. Other valuable chemicals might also be produced from CO_2_-derived acetate by introducing different metabolic pathways into *P. stutzeri* (∆*pst_3217*).

Acetate is much cheaper than glucose and many other conventional carbon sources. However, it is also highly toxic to most of industrial microorganisms. Thus, researchers developed many strategies to enhance the microbial acetate tolerance. For example, Seong et al. (2020) conducted adaptive laboratory evolution of *E. coli* for its efficient consumption and tolerance to acetate. Upregulated expression of genes related to ATP biosynthesis and high intracellular ATP concentrations are the possible explanations for enhanced acetate utilization of the evolved strain. In this study, the genes involved in acetyl-CoA generation through L-leucine catabolism were deleted in *P. stutzeri* A1501 and the growth and tolerance of the recombinant *P. stutzeri* (∆*pst_3217*) with acetate were obviously improved. To identify the detailed mechanism of improved tolerance of *P. stutzeri* (*∆pst_3217*), multiomics analysis of *P. stutzeri* (∆*pst_3217*) during acetate utilization is required. The effects of *pst_3217* deletion and overexpression of the key enzymes like acetate kinase, phosphotransacetylase and acetyl-CoA synthetase on acetate utilization of *P. stutzeri* A1501 should also be compared.

Interestingly, the mutant strain *P. stutzeri* (*Δpst_1727Δpst_2502*), in which the acetyl-CoA production from L-isoleucine was blocked, also exhibited a shorter lag phase in acetate (Supplementary Figure S5). In addition, the mutant with deletion of pyruvate dehydrogenase (*aceEF*) in *E. coli* K12 (DE3) was reported to display faster rate of acetate consumption in MM medium with acetate as the only carbon source (Da et al., 2021). Since acetate can inhibit microbial growth and decrease metabolic efficiency, the catabolism of intracellular substances like amino acids and pyruvate may participate in supporting the survival of the microorganisms during the lag period in medium with acetate. However, these endogenous acetyl-CoA production processes may interfere with the acetyl-CoA production from exogenous acetate. Thus, we supposed that the improved acetate tolerance and cell growth after blocking these pathways may be the results of decreased endogenous acetyl-CoA production and possibly increased acetyl-CoA generation from exogenous acetate. Overexpression of the key enzymes for acetate activation like acetate kinase, phosphotransacetylase and acetyl-CoA synthetase in the mutant strains mentioned above may further improve their robustness and efficiency of acetate assimilation, and thus is worth trying in subsequent researches.

## Conclusion

In this study, blocking of endogenous acetyl-CoA production through l-leucine catabolism was found to be beneficial for the growth of *P. stutzeri* A1501 with acetate. *P. stutzeri* (Δ*pst_3217*), which grew faster than other mutants, was applied to produce PHB from acetate through overexpressing *phbCAB* from *R. eutropha* H16. PHB accounted for 5.42% (w/w) of dried cell weight of *P. stutzeri* (∆*pst_3217*-*phbCAB*) was produced from CO_2_-derived acetate. This study identified the feasibility of improving acetate utilization through blocking endogenous acetyl-CoA generation and other strains with enhanced acetate utilization may also be constructed using similar strategy.

## Data Availability

The original contributions presented in the study are included in the article/Supplementary Material, further inquiries can be directed to the corresponding author.
