# Supplementary material for: Poly-3-hydroxybutyrate production from acetate by recombinant Pseudomonas stutzeri with blocked L-leucine catabolism and enhanced growth in acetate
Source: Front Bioeng Biotechnol. 2023 Nov 8;11:1297431. doi: 10.3389/fbioe.2023.1297431 (PMC10663377; doi:10.3389/fbioe.2023.1297431)
Supplement: Supplementary file 1 [file DataSheet1.docx]

**Poly-3-hydroxybutyrate production from acetate by recombinant *Pseudomonas stutzeri* with blocked l-leucine catabolism and enhanced growth in acetate**

**Jieni Zhu^1^,** **Wei Liu^1^,** **Mengjiao Wang^1^,** **Haiyan Di^1^, Chuanjuan Lü^1^, Ping Xu^2^, Chao Gao^1^, Cuiqing Ma^1^***

^1^*State Key Laboratory of Microbial Technology, Shandong University, Qingdao 266237, China*

*^2^State Key Laboratory of Microbial Metabolism, Shanghai Jiao Tong University, Shanghai 200240, China*

*** Corresponding:**

Cuiqing Ma

macq@sdu.edu.cn

**Keywords:** acetate, *Pseudomonas stutzeri*, l-leucine catabolism, poly-3-hydroxybutyrate, CO_2_


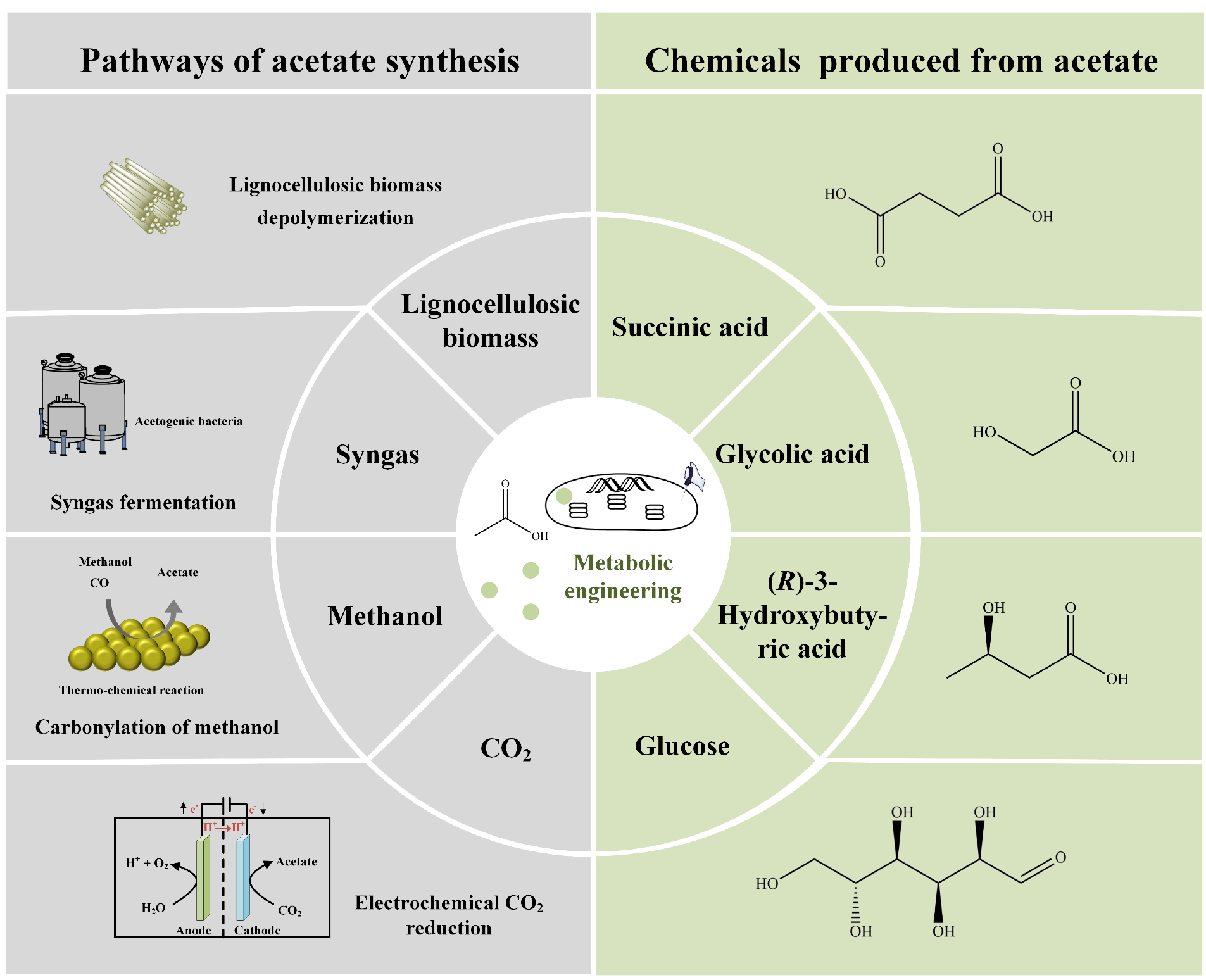


**Figure S1.** Pathways of acetate synthesis and chemicals produced from acetate by metabolic engineered microorganisms.


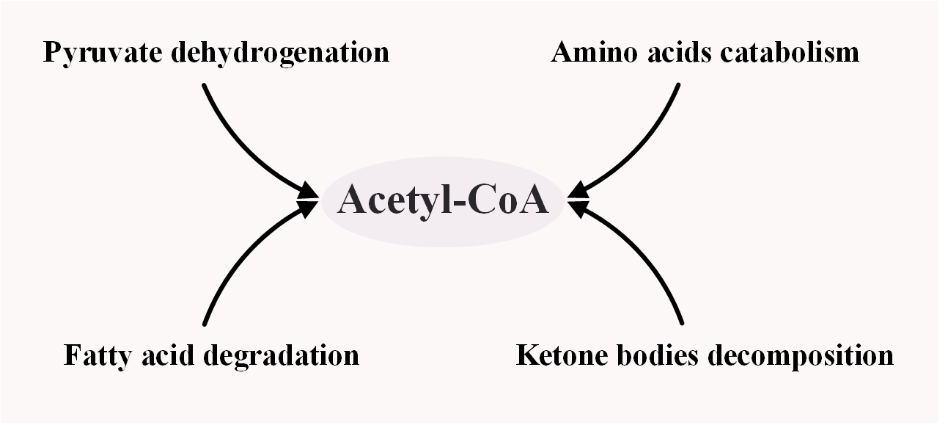


**Figure S2.** Endogenous metabolic pathways of acetyl-CoA generation.


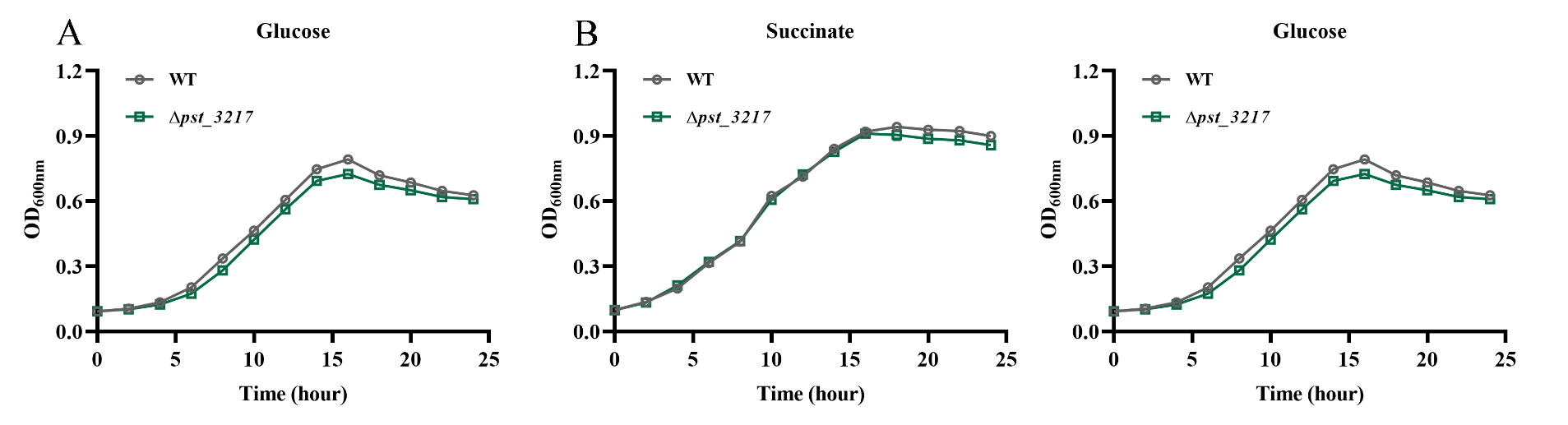


**Figure** **S3.** Growth of *P. stutzeri* A1501 and *P. stutzeri* (∆*pst_3217*) in AB minimal medium containing glucose and succinate. **(A**) The growth of *P. stutzeri* A1501 and *P. stutzeri* (∆*pst_3217*) in AB minimal medium with 20 mM glucose. **(B**) The growth of *P. stutzeri* A1501 and *P. stutzeri* (∆*pst_3217*) in AB minimal medium with 20 mM succinate.


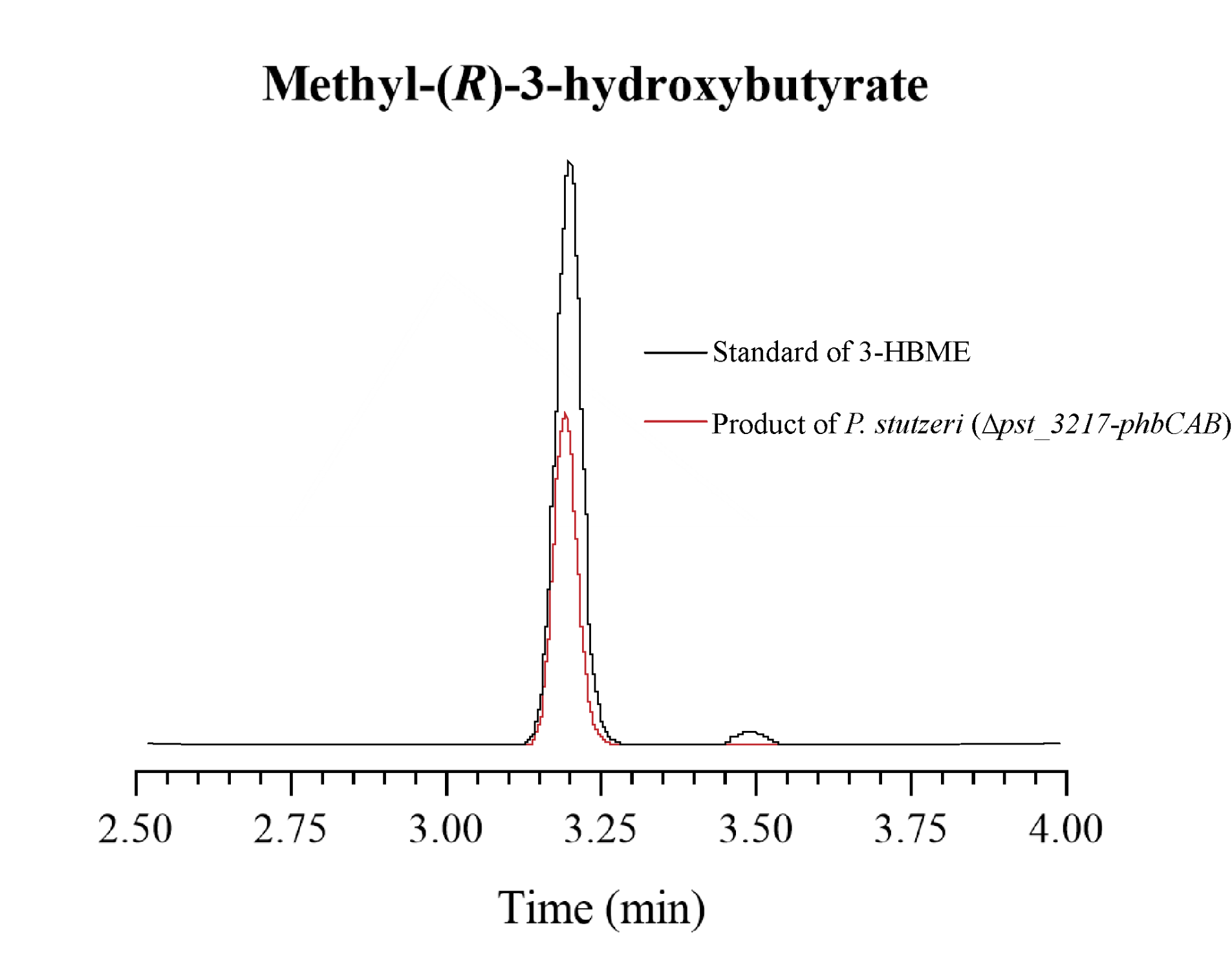


**Figure S4.** Gas chromatography analysis of 3-hydroxybutyrate methyl ester (3-HBME) standard and product of *P. stutzeri* (∆*pst_3217*-*phbCAB*) after methanol esterification.


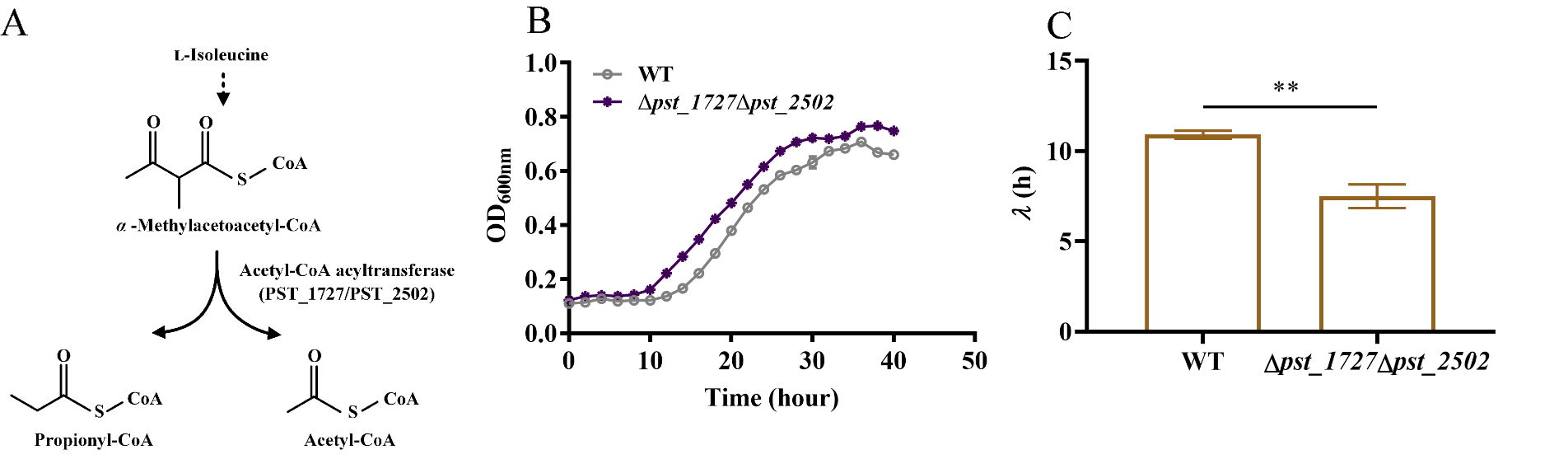


**Figure S5.** Deletion of *pst_1727* and *pst_2502* reduced lag time of *P. stutzeri* A1501 with acetate. **(A)** The reaction catalyzed by acetyl-CoA acyltransferase (PST_1727 and PST_2502). **(B)** Growth of *P. stutzeri* A1501 and *P. stutzeri* (∆*pst_1727*Δ*pst_2502*) in AB minimal medium with 40 mM acetate. **(C)** The lag time (*λ*) of *P. stutzeri* A1501 and *P. stutzeri* (∆*pst_1727*Δ*pst_2502*) in AB minimal medium with 40 mM acetate. All data shown are the average values of three independent experiments.

**Table S1.** Strains and plasmids used in this study.

| **Strain or plasmid** | **Description^a^** | **Source** |
| --- | --- | --- |
| **Strain** |  |  |
| *P. stutzeri* A1501 | Wild-type | Laboratory stock |
| *P. stutzeri* (∆*pst_3213*) | *P. stutzeri* A1501 with a deletion of *pst_3213* gene | This study |
| *P. stutzeri* (∆*pst_3214*) | *P. stutzeri* A1501 with a deletion of *pst_3214* gene | This study |
| *P. stutzeri* (∆*pst_3215*) | *P. stutzeri* A1501 with a deletion of *pst_3215* gene | This study |
| *P. stutzeri* (∆*pst_3216*) | *P. stutzeri* A1501 with a deletion of *pst_3216* gene | This study |
| *P. stutzeri* (∆*pst_3217*) | *P. stutzeri* A1501 with a deletion of *pst_3217* gene | This study |
| *P. stutzeri* (∆*pst_3218*) | *P. stutzeri* A1501 with a deletion of *pst_3218* gene | This study |
| *P. stutzeri* (∆*pst_3219*) | *P. stutzeri* A1501 with a deletion of *pst_3219* gene | This study |
| *P. stutzeri* (∆*pst_1727*) | *P. stutzeri* A1501 with a deletion of *pst_1727* gene | This study |
| *P. stutzeri* (∆*pst_1727*∆*pst_2502*) | *P. stutzeri* A1501 with deletions of *pst_1727* gene and *pst_2502* gene | This study |
| *P. stutzeri* (∆*pst_3217*-pBBR) | *P. stutzeri* (∆*pst_3217*) harboring the plasmid pBBR1MCS-2 | This study |
| *P. stutzeri* (∆*pst_3217-phbCAB*) | *P. stutzeri* (∆*pst_3217*) harboring the expression plasmid pBBR1MCS-2*-phbCAB* | This study |
| *E. coli* DH5*α* | F^–^ φ80*lacZ*∆M15 ∆(*lacZYA-argF*)U169 *deoR recA*1 *endA*1 *hsdR*17(r_K_^–^, m_K_^+^) *phoA supE*44 λ^–^ *thi-*1 *gyrA*96 *relA*1, used for gene clone | Invitrogen |
| *E. coli* HB101 | F^–^ *mcrB mrr hsdS20*(r_B_^–^m_B_^–^) *recA13 supE44 ara14 proA2 lacY1* *galK2 xy15 λ*^-^ *mtl1 rpsL20*(Sm^r^) *glnV44* λ^-^, triparental mating helper strain | Invitrogen |
| *R. eutropha* H16 | Wild-type | Laboratory stock |
| **Plasmid** |  |  |
| pBBR1MCS-2 | Vector for gene expression, Km^r^ | Laboratory stock |
| pBBR1MCS-2-*phbCAB* | pBBR1MCS-2 with *phbCAB* from *R. eutropha* H16, Km^r^ | This study |
| pRK2013 | Helper plasmid for conjugation, Km^r^ | Invitrogen |
| pK18*mobsacB* | Suicide plasmid for gene knockout, Km^r^ and Tc^r^ | Schäfer, et al.,1994 |
| pK18*mobsacB*-*pst_3213**’* | Partial lengths of *pst_3213* were inserted into pK18*mobsacB*, Km^r^ and Tc^r^ | This study |
| pK18*mobsacB*-*pst_3214’* | Partial lengths of *pst_3214* were inserted into pK18*mobsacB*, Km^r^ and Tc^r^ | This study |
| pK18*mobsacB*-*pst_3215’* | Partial lengths of *pst_3215* were inserted into pK18*mobsacB*, Km^r^ and Tc^r^ | This study |
| pK18*mobsacB*-*pst_3216’* | Partial lengths of *pst_3216* were inserted into pK18*mobsacB*, Km^r^ and Tc^r^ | This study |
| pK18*mobsacB*-*pst_3217’* | Partial lengths of *pst_3217* were inserted into pK18*mobsacB*, Km^r^ and Tc^r^ | This study |
| pK18*mobsacB*-*pst_3218’* | Partial lengths of *pst_3218* were inserted into pK18*mobsacB*, Km^r^ and Tc^r^ | This study |
| pK18*mobsacB*-*pst_3219’* | Partial lengths of *pst_3219* were inserted into pK18*mobsacB*, Km^r^ and Tc^r^ | This study |
| pK18*mobsacB*-*pst_1727’* | Partial lengths of *pst_1727* were inserted into pK18*mobsacB*, Km^r^ and Tc^r^ | This study |
| pK18*mobsacB*-*pst_2502’* | Partial lengths of *pst_2502* were inserted into pK18*mobsacB*, Km^r^ and Tc^r^ | This study |

^a^Km^r^, kanamycin resistant; Tc^r^, tetracycline resistant.

**Table S2.** The primers used in this study.

| **Primer** | **Sequence (5’-3’)** |
| --- | --- |
| uf-*pst_3213* | TATGACATGATTACGAATTCATGGCGATACATGCAGGCGAAGC |
| ur-*pst_3213* | GTTGTACCGCCCGCAGCGGGGCCACCTGCTTGTCTTGTAATT |
| df-*pst_3213* | GACAAGCAGGTGGCCCCGCTGCGGGCGGTACAACGCCCAACT |
| dr-*pst_3213* | CGCTAACGGATTCAGGATCCGCGACGTCTTCGCCATAGACCTCAT |
| uf-*pst_3214* | TATGACATGATTACGAATTCCATGCAGGCCTGCCTCGACG |
| ur-*pst_3214* | CTGCCGGCGGCACGGAGGTTGGCTCCGTTCAATT |
| df-*pst_3214* | TTGAACGGAGCCAACCTCCGTGCCGCCGGCAGCC |
| dr-*pst_3214* | CGCTAACGGATTCAGGATCCATCACCGCCGGCGCCAGG |
| uf-*pst_3215* | TATGACATGATTACGAATTCGCATCGCCAAGCACGGCGCCAA |
| ur-*pst_3215* | TCGATATGGCGATTCACTCGGCATCCTCGATGCGACTGC |
| df-*pst_3215* | CATCGAGGATGCCGAGTGAATCGCCATATCGATACCCTGCT |
| dr-*pst_3215* | CGCTAACGGATTCAGGATCCGCCGCCACCGGCGGTGG |
| uf-*pst_3216* | TATGACATGATTACGAATTCCTGCCGCAGCCGACCCT |
| ur-*pst_3216* | CCACCTTGTGCATCCGCACTTCTTCTCCTGCCAGGCG |
| df-*pst_3216* | GGCAGGAGAAGAAGTGCGGATGCACAAGGTGGACAAGGC |
| dr-*pst_3216* | CGCTAACGGATTCAGGATCCATGTTCTTCTGCGAAAAAGCTTC |
| uf-*pst_3217* | ATGACATGATTACGAATTCGCATTGATCGTGCTGGAAGCGATGAAGATGG |
| ur-*pst_3217* | ACCCGCGAACCGTTGGCACGACGCGGGCCGACTTCCACCA |
| df-*pst_3217* | TCGTGCCAACGGTTCGCGGGTCGCCCGCGCCAGGCATGCCGGTTA |
| dr-*pst_3217* | CGCTAACGGATTCAGGATCCCTGATCTGCCGCGTCTCCAGCAGCAGGC |
| uf-*pst_3218* | TATGACATGATTACGAATTCGGCCCGCGAGCTGTATGCCATG |
| ur-*pst_3218* | TGTCCAGGCCATGGTCGAATTACCTCTACTTGTTGT |
| df-*pst_3218* | CAAGTAGAGGTAATTCGACCATGGCCTGGACACGTGA |
| dr-*pst_3218* | CGCTAACGGATTCAGGATCCCGAGGCCGGTGAGCGGCAGGT |
| uf-*pst_3219* | TATGACATGATTACGAATTCGACGCGGCAGATCAGCAAGA |
| ur-*pst_3219* | TTCCCGCACCCAGGCTGGTCTCAGCTCCTCACCGTGCGTTT |
| df-*pst_3219* | GTGAGGAGCTGAGACCAGCCTGGGTGCGGGAAGCA |
| dr-*pst_3219* | CGCTAACGGATTCAGGATCCGGACGGAACGGCGTGGG |
| uf-*pst_1727* | TATGACATGATTACGAATTCCGCCTCGGTCAGAAGAACGGCAAG |
| ur-*pst_1727* | CATCTGCATCTGTCTGGAAATCTCTCTCCCTCGATTAACCGTAG |
| df-*pst_1727* | AGGGAGAGAGATTTCCAGACAGATGCAGATGACTGCGCTA |
| dr-*pst_1727* | CGCTAACGGATTCAGGATCCCGCTATATATAGTGGCGATGGA |
| uf-*pst_2502* | TATGACATGATTACGAATTCTGGCTGATATGGTCGACCGCAC |
| ur-*pst_2502* | GCAGCTTCGAGGGCGGACATACCTCGGAGACTAGGCG |
| df-*pst_2502* | TCTCCGAGGTATGTCCGCCCTCGAAGCTGCTCACAGAAA |
| dr-*pst_2502* | CGCTAACGGATTCAGGATCCCCTTCGCTCTGGCCAAGCACTT |
| *phbCAB*-f | ATATCGAATTCCTGCAGCCCGGGCAAGTACCTTGCCGACA |
| *phbCAB*-r | CTAGAACTAGTGGATCCCCCCTTCTGAATCCATGACCAGC |

**References**

Schäfer, A., Tauch, A., Jäger, W., Kalinowski, J., Thierbach, G., and Pühler, A. (1994). Small mobilizable multi-purpose cloning vectors derived from the *Escherichia coli* plasmids pK18 and pK19: selection of defined deletions in the chromosome of *Corynebacterium glutamicum*. *Gene* 145, 69–73. doi: 10.1016/0378-1119(94)90324-7
